# IgG_4_ inhibits peanut-induced basophil and mast cell activation in peanut-tolerant children sensitized to peanut major allergens

**DOI:** 10.1016/j.jaci.2015.01.012

**Published:** 2015-05

**Authors:** Alexandra F. Santos, Louisa K. James, Henry T. Bahnson, Mohammed H. Shamji, Natália C. Couto-Francisco, Sabita Islam, Sally Houghton, Andrew T. Clark, Alick Stephens, Victor Turcanu, Stephen R. Durham, Hannah J. Gould, Gideon Lack

**Affiliations:** a Department of Pediatric Allergy, Division of Asthma, Allergy & Lung Biology, King's College London, London, United Kingdom; b MRC & Asthma UK Centre in Allergic Mechanisms of Asthma, London, United Kingdom; c Immunoallergology Department, Coimbra University Hospital, Coimbra, Portugal; d Gulbenkian Programme for Advanced Medical Education, Lisbon, Portugal; e Randall Division of Cell and Molecular Biophysics, King's College London, London, United Kingdom; f Independent Biostatistician, Chapel Hill, NC; g Allergy and Clinical Immunology, MRC & Asthma UK Centre for Allergic Mechanisms of Asthma Faculty of Medicine, National Heart and Lung Institute, Imperial College London, London, United Kingdom; h Department of Medicine, University of Cambridge, Addenbrooke's Hospital, Cambridge, United Kingdom; i Pathology Partnership at Cambridge University Hospitals, Cambridge, United Kingdom; j Department of Allergy, Cambridge University Hospitals NHS Foundation Trust, Addenbrooke's Hospital, Cambridge, United Kingdom

**Keywords:** Ara h 2, basophil, basophil activation test, blocking antibodies, IgE inhibition, IgG_4_, mast cells, peanut, peanut allergy, tolerance, HSA, Human serum albumin, NA, Non–peanut-sensitized nonallergic, PA, Peanut allergy, POIT, Peanut oral immunotherapy, P-sIgE, Peanut-specific IgE, P-sIgG_4_, Peanut-specific IgG_4_, PS, Peanut-sensitized but tolerant, SPT, Skin prick test

## Abstract

**Background:**

Most children with detectable peanut-specific IgE (P-sIgE) are not allergic to peanut. We addressed 2 non–mutually exclusive hypotheses for the discrepancy between allergy and sensitization: (1) differences in P-sIgE levels between children with peanut allergy (PA) and peanut-sensitized but tolerant (PS) children and (2) the presence of an IgE inhibitor, such as peanut-specific IgG_4_ (P-sIgG_4_), in PS patients.

**Methods:**

Two hundred twenty-eight children (108 patients with PA, 77 PS patients, and 43 nonsensitized nonallergic subjects) were studied. Levels of specific IgE and IgG_4_ to peanut and its components were determined. IgE-stripped basophils or a mast cell line were used in passive sensitization activation and inhibition assays. Plasma of PS subjects and patients submitted to peanut oral immunotherapy (POIT) were depleted of IgG_4_ and retested in inhibition assays.

**Results:**

Basophils and mast cells sensitized with plasma from patients with PA but not PS patients showed dose-dependent activation in response to peanut. Levels of sIgE to peanut and its components could only partially explain differences in clinical reactivity between patients with PA and PS patients. P-sIgG_4_ levels (*P* = .023) and P-sIgG_4_/P-sIgE (*P* < .001), Ara h 1–sIgG_4_/Ara h 1–sIgE (*P* = .050), Ara h 2–sIgG_4_/Ara h 2–sIgE (*P* = .004), and Ara h 3–sIgG_4_/Ara h 3–sIgE (*P* = .016) ratios were greater in PS children compared with those in children with PA. Peanut-induced activation was inhibited in the presence of plasma from PS children with detectable P-sIgG_4_ levels and POIT but not from nonsensitized nonallergic children. Depletion of IgG_4_ from plasma of children with PS (and POIT) sensitized to Ara h 1 to Ara h 3 partially restored peanut-induced mast cell activation (*P* = .007).

**Conclusions:**

Differences in sIgE levels and allergen specificity could not justify the clinical phenotype in all children with PA and PS children. Blocking IgG_4_ antibodies provide an additional explanation for the absence of clinical reactivity in PS patients sensitized to major peanut allergens.

Sensitization is the first stage in the development of IgE-mediated food allergy. Clinically, it is demonstrated by a positive skin prick test (SPT) response or detectable allergen-specific IgE antibodies in serum. However, allergic sensitization does not always lead to clinical allergy. In fact, the majority of subjects with detectable food-specific IgE do not have any allergic symptoms when consuming the food.^1,2^ In other words, allergen-specific IgE is necessary but not sufficient for the development of immediate-type food allergy.

The discrepancy between allergic sensitization and clinical food allergy creates diagnostic difficulties and a fundamental gap in our knowledge about the mechanisms of food allergy and tolerance. We have used peanut allergy (PA) as a model of IgE-mediated food allergy. Specific IgE to Ara h 2 is particularly discriminative in identifying patients with PA.^1,3^ However, examples can be found of peanut-tolerant patients who have high levels of Ara h 2–specific IgE and conversely of patients with PA who have negative test results to Ara h 2 and the other major peanut allergens.^4^ Basophils and mast cells are the effector cells of acute allergic reactions to foods, including anaphylaxis. In a previous study we showed that a whole-blood basophil activation test has high accuracy in the diagnosis of PA and correlates very closely with the clinical phenotype of IgE-sensitized patients (ie, allergic vs tolerant patients) at a level better than measurement of specific IgE levels to peanut, Ara h 2, or any of the other peanut components.^5^ Similar *in vitro* systems using passive sensitization of basophils or mast cells with patients' plasma can be used to test the ability of allergen-specific IgE antibodies present in the plasma to elicit effector cell activation and degranulation in response to the allergen.

In this study we addressed 2 non–mutually exclusive hypotheses to explain the discrepancy between allergic sensitization and clinical allergy. The first hypothesis was that the levels and specificity of IgE are different between allergic and tolerant patients. The second hypothesis was that sensitized but tolerant patients have an inhibitor that blocks the function of IgE. Given that natural tolerance to food allergens is allergen specific and long-lasting, the IgE inhibitor is likely to be a food-specific antibody of an isotype other than IgE, such as IgG_4_. IgG_4_ levels have been shown to increase in patients who naturally outgrow IgE-mediated food allergy, such as cow's milk allergy,^6,7^ and in patients who are submitted to food oral immunotherapy^8,9^ and immunotherapy to respiratory allergens.^10–12^ Whether IgG_4_ can play an inhibitory role in the allergen-IgE interaction in sensitized but otherwise tolerant patients is unknown. IgG_4_ is produced as part of a T_H_2-type immune response induced mainly by the tolerogenic cytokine IL-10^13^ and therefore was the main suspect for being the IgE inhibitor in peanut-sensitized but tolerant (PS) patients in this study.

## Methods

### Study population

Children with PA, PS children, and non–peanut-sensitized nonallergic (NA) children consecutively attending pediatric allergy clinics at a university hospital or a private hospital in London were invited to participate in the study. Patients were clinically evaluated, including oral food challenges to peanut, if clinically indicated and as previously described.^5^ The patient's allergic status to peanut was determined by using oral food challenges, except for (1) children with a convincing history of a systemic reaction or reactions to peanut within 1 year of their visit and an SPT-induced wheal size of 8 mm or greater,^8^ a peanut-specific IgE (P-sIgE) level of 15 KU_A_/L or greater,^8^ or both, who were considered to have PA, and (2) children who were able to eat 4 g or more of peanut protein twice a week (as assessed by a validated peanut consumption questionnaire^14^) without having allergic symptoms, who were considered peanut tolerant. Peanut sensitization was defined as an SPT-induced wheal size of 1 mm or greater, a P-sIgE level of 0.10 KU_A_/L or greater, or both. Serum and plasma samples were collected simultaneously for serology and for subsequent mast cell and basophil passive sensitization experiments, respectively. The parents of all children signed an informed consent form approved by the South East London Research Ethics Committee 2.

Plasma samples collected before and after treatment from an independent population of 19 patients with PA who underwent peanut oral immunotherapy (POIT) as part of the STOP I trial (registered at http://ClinicalTrials.gov with the identification no. NCT01259804)^15^ were tested in parallel.

### Serum specific IgE and IgG_4_ levels to peanut and peanut components

Serum specific IgE and IgG_4_ to peanut extract and to the recombinant peanut allergens rAra h 1, rAra h 2, rAra h 3, rAra h 8, and rAra h 9 were measured with an immunoenzymatic assay (ImmunoCAP; Thermo Fisher, Waltham, Mass). IgG_4_/IgE ratios were determined after conversion of kilounits per liter (IgE) and milligrams per liter (IgG_4_) to nanograms per milliliter.

### IgG_4_ antibody depletion

IgG_1_ anti-IgG_4_ antibody (clone MH164-4; Sanquin, Amsterdam, The Netherlands) was coupled to cyanogen bromide–activated Sepharose (GE Healthcare, Hertfordshire, United Kingdom) during an overnight incubation at 4°C. The remaining reactive groups were blocked with 1 mol/L ethanolamine, followed by 3 cycles of washes in alternating pH using 0.1 mol/L acetic acid/sodium acetate at pH 4.0 and 0.1 mol/L Tris-HCl at pH 8.0. Mock-coupled Sepharose beads were processed in parallel with anti-IgG_4_–coupled beads. Plasma samples were filtered and diluted 1:10 in PBS-AT (0.3% BSA, 0.1% Tween 20, and 0.05% NaN_3_ in PBS). Diluted plasma samples were incubated with anti-IgG_4_– or mock-coupled Sepharose beads in a total volume of 500 μL overnight at room temperature with continuous end-over-end rotation. IgG_4_- and mock-depleted samples were collected by means of centrifugation. Total IgG_4_ plasma levels were measured by means of ELISA in IgG_4_- and mock-depleted samples, as previously described.^16^

### Basophil and mast cell activation and inhibition assays

For the basophil assays, PBMCs were isolated from citrate-dextrose–anticoagulated blood of atopic non–peanut-sensitized adult volunteers without peanut allergy by using density gradient separation with Histopaque-1077 (Sigma-Aldrich, Poole, United Kingdom). For stripping of receptor-bound IgE, PBMCs were resuspended in lactic acid (13.4 mmol/L lactate, 140 mmol/L NaCl, and 5 mmol/L KCl [pH 3.9]) and incubated at 4°C for 5 minutes. Human serum albumin (HSA; 0.5%) in RPMI was added, and the solution was neutralized with 12% Tris. After washing with 0.5% HSA, the cell pellet was resuspended in 0.5% HSA and incubated for 1 hour at 37°C with individual plasma from study participants. After washing, resensitized PBMCs were stimulated with peanut extract (10 ng/mL; ALK-Abelló, Hørsholm, Denmark), anti-IgE (1 μg/mL, Sigma-Aldrich), formyl-methionyl-leucyl-phenylalanine (1 μmol/L, Sigma-Aldrich), or 0.5% HSA alone for 1 hour at 37°C and stained with CD123–fluorescein isothiocyanate (eBioscience, San Diego, Calif), HLA-DR–peridinin-chlorophyll-protein, CD203c-phycoerythrin, and CD63-allophycocyanin (BioLegend, San Diego, Calif), followed by flow cytometric analysis. Basophils were gated as side scatter–low CD203c^+^ CD123^+^HLA-DR^−^ cells. The peanut extract used contained all 3 major peanut allergens, Ara h 1, Ara h 2, and Ara h 3, as previously reported.^5^ Basophil activation was expressed as the percentage of CD63^+^ basophils and as the stimulation index of CD203c.

For the mast cell assays, LAD2 cells,^17^ which were kindly provided by Drs Dean Metcalfe and Arnold Kirshenbaum (Laboratory of Allergic Diseases, National Institute of Allergy and Infectious Diseases), were placed in culture with rIL-4 for 5 days before overnight sensitization with patients' plasma. Sensitized cells were stimulated with peanut extract, anti-IgE, ionomycin, or 0.04% BSA RPMI alone for 30 minutes at 37°C and stained with the viability dye eFluor 450 and CD107b–fluorescein isothiocyanate (eBioscience), CD203c-phycoerythrin, CD107a-PerCPCy5.5, CD63-allophycocyanin, and IgE-PECy7 (BioLegend) and analyzed by means of flow cytometry. LAD2 cells were gated as CD203c^+^ viable cells. Mast cell activation was expressed as the percentage of CD63^+^ or CD107a^+^ cells.

For mast cell inhibition experiments, 20 μL of plasma from PS patients or NA subjects or stimulation buffer was incubated with an equal volume of stimulation buffer and 10 μL of allergen at 37°C for 1 hour before LAD2 cells previously sensitized with 100 μL of plasma from a patient with PA and washed were added to the allergen-plasma mixture. For basophil inhibition experiments, the 20 μL of plasma from a patient with PA was incubated with 20 μL of plasma from PS patients or NA subjects and allergen before addition of 1 million IgE-stripped PBMCs. The same plasma from patients with PA was used in all mast cell inhibition experiments. Another plasma sample with similar IgE levels and patterns of sensitization to peanut allergens (ie, positive to Ara h 1, Ara h 2, and Ara h 3) was used in all basophil inhibition experiments.

Flow cytometry was performed on a FACSCanto II with FACSDiva software (BD Biosciences, San Jose, Calif), and data were analyzed with FlowJo software (version 7.6.1; TreeStar, Ashland, Ore).

### Statistical analyses

Qualitative variables were compared between children with PA and PS children by using Fisher exact or χ^2^ tests, and continuous variables were compared by using the Mann-Whitney *U* or Kruskall-Wallis tests. For paired analyses, the Wilcoxon signed-rank test was used. Statistical analyses were performed with SPSS 20.0 (SPSS, Chicago, Ill) and JMP Pro 11.2.1 software for Windows. Significance was determined by using a 2-sided α level of .05.

## Results

### Study population

Two hundred twenty-eight children (108 children with PA and 110 peanut-tolerant children [77 PS and 43 NA children]) were included in the study. Children with PA were slightly older (median, 6 years) and more frequently had asthma (39.8%) and allergic rhinitis (59.3%) than PS children (median, 4 years; 18.2% with asthma and 31.2% with allergic rhinitis). The other demographic and clinical features were similar between the 2 groups (Table I).

### Passive sensitization basophil and mast cell assays reproduced *in vitro* clinical reactivity to peanut *in vivo*

**Fig E1:**
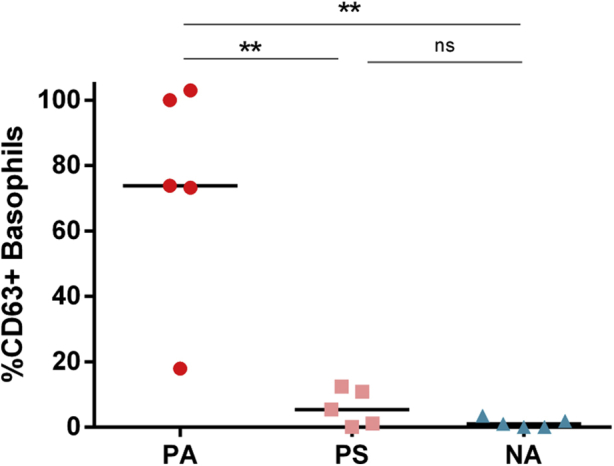


**Fig 1:**
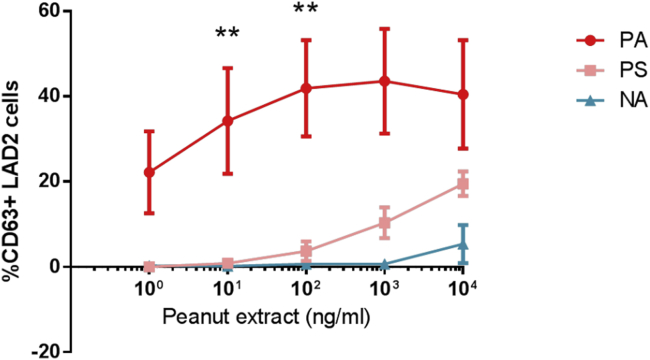
Peanut-induced activation of mast cells (expressed as a percentage of CD63^+^ LAD2 cells) sensitized with plasma from patients with PA (n = 6), PS patients (n = 5), and NA subjects (n = 2). Similar results were found for mast cell activation expressed as a percentage of CD107a^+^ LAD2 cells. Values are presented as means and SEs. *P* values refer to the comparison between patients with PA and PS patients for each concentration of peanut extract using the Mann-Whitney *U* test. **P < .01.

In a previous study we showed that a whole-blood basophil activation test reproduces very closely the clinical phenotype of peanut-sensitized patients.^5^ Here we performed similar experiments in which LAD2 cells or IgE-stripped primary basophils from healthy donors were sensitized with plasma from patients with PA, PS patients, or NA subjects before stimulation with peanut extract. Mast cells sensitized with plasma from patients with PA, but not from PS patients or NA subjects, showed a dose-dependent activation in response to peanut (Fig 1). The same findings were observed with primary human basophils (see Fig E1 in this article's Online Repository at www.jacionline.org).

### Levels of specific IgE to peanut and peanut components only partially account for differences in clinical reactivity to peanut between patients with PA and PS patients

**Fig E2:**
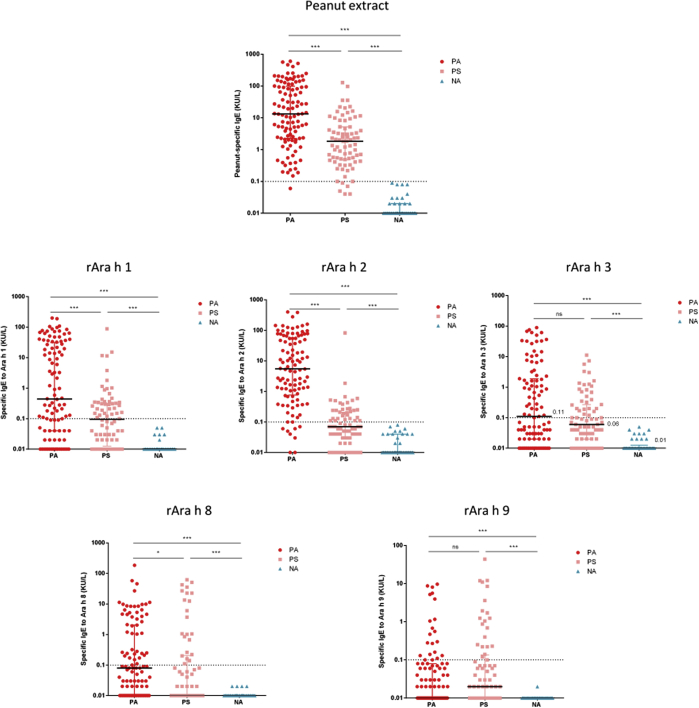


Patients with PA had higher levels of IgE to peanut (*P* < .001) and Ara h 1 (*P* < .001), Ara h 2 (*P* < .001), and Ara h 8 (*P* = .019) than PS patients (Table I). However, there was a substantial overlap between the 2 groups (see Fig E2 in this article's Online Repository at www.jacionline.org).

There was no obvious relationship between the profiles of IgE sensitization to peanut allergens and clinical reactivity (Table II and see Table E1 in this article's Online Repository at www.jacionline.org). The majority (69%) of patients with PA were sensitized to more than 1 peanut allergen, whereas PS patients were more likely to be sensitized to 1 or none of the peanut major allergens (61%). Although there was a substantial overlap between patients with PA and PS patients, there were 2 unique patterns of sensitization: all patients sensitized simultaneously to Ara h 1 and to Ara h 2 had PA, and all patients monosensitized to Ara h 1 were PS.

### Peanut-specific IgG_4_ levels were greater in PS patients compared with those in patients with PA

**Fig E3:**
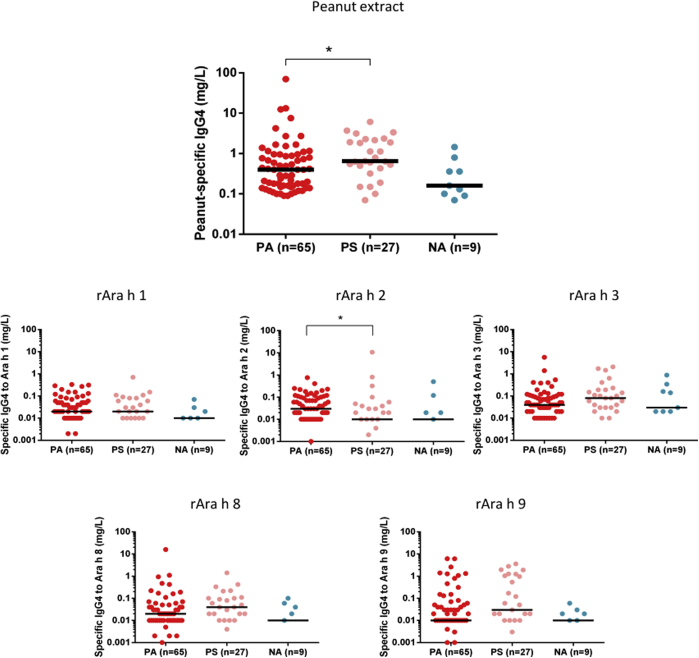


One hundred one patients (65 patients with PA, 27 PS patients, and 9 NA subjects) with detectable peanut-specific IgG_4_ (P-sIgG_4_) levels were tested for IgG_4_ to peanut components. P-IgG_4_ levels were 1.6-fold higher in PS patients than in patients with PA (*P* = .012, see Fig E3 in this article's Online Repository at www.jacionline.org). However, there were no significant differences between the levels of specific IgG_4_ to peanut components in patients with PA and PS patients (Table III), except for Ara h 2–specific IgG_4_ levels, which were greater in patients with PA (*P* = .034).

### P-sIgG_4_/P-sIgE ratio was significantly greater in sensitized but tolerant patients

**Fig E4:**
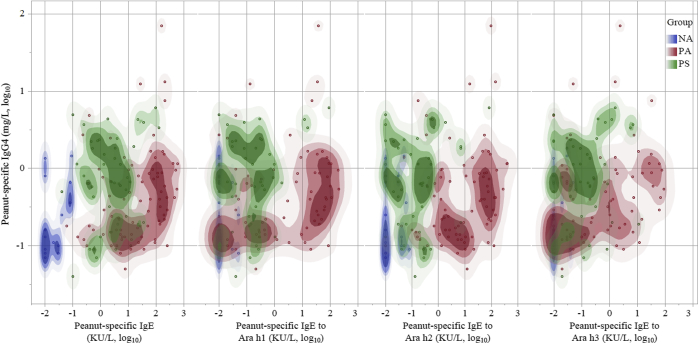


**Fig 2:**
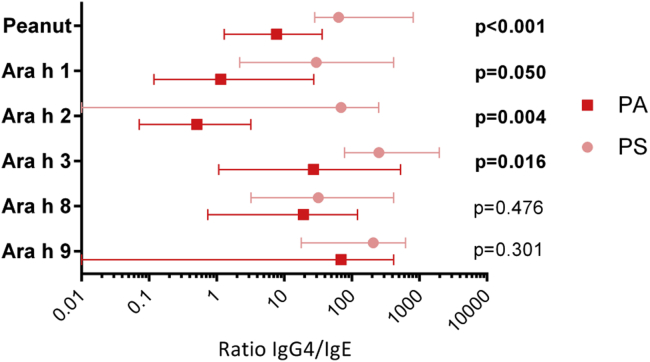
IgG_4_/IgE ratios to peanut and peanut allergens in children with PA (n = 65) and PS children (n = 27). Values are presented as medians and interquartile ranges. *P* values refer to the comparison between children with PA and PS children using the Mann-Whitney *U* test.

Overall, the levels and specificity of IgG_4_ to peanut could not account for the clinical differences between patients with PA and PS patients. However, the ratio of specific IgG_4_ to IgE directed to peanut was 8 times higher in PS patients compared with that in patients with PA (Fig 2). The differences between PS patients and patients with PA were even greater in the IgG_4_/IgE ratio to Ara h 1 (18.8-fold, *P* = .05), Ara h 2 (100-fold, *P* = .004), and Ara h 3 (7-fold, *P* = .016). In patients with PA submitted to POIT (n = 19), the levels of P-sIgE were similar before and after treatment, but the levels of P-sIgG_4_ and the P-sIgG_4_/P-sIgE ratio were 12-fold and 10-fold higher after immunotherapy, respectively (see Table E2 in this article's Online Repository at www.jacionline.org). Differences in P-sIgG_4_/P-sIgE ratios between patients with PA and PS patients remained significant after adjusting for specific IgE levels by using analysis of covariance with ranks (*P* = .001). Similarly, by using a multivariate logistic regression model, log base 10–transformed IgG_4_ (*P* = .004) and IgE (*P* < .001) levels were both significantly associated with peanut allergy. A relative importance analysis showed that IgE accounted for 64% of the model's explanatory power in predicting patients with PA versus PS patients, and IgG_4_ explained the remaining 36%. Patients with PA tended to have higher levels of IgE to peanut and peanut major allergens, and PS patients showed a predominance of IgG_4_ over IgE (see Fig E4 in this article's Online Repository at www.jacionline.org).

### Plasma from PS patients was able to inhibit peanut-induced basophil and mast cell activation similar to plasma from patients submitted to POIT

**Fig E5:**
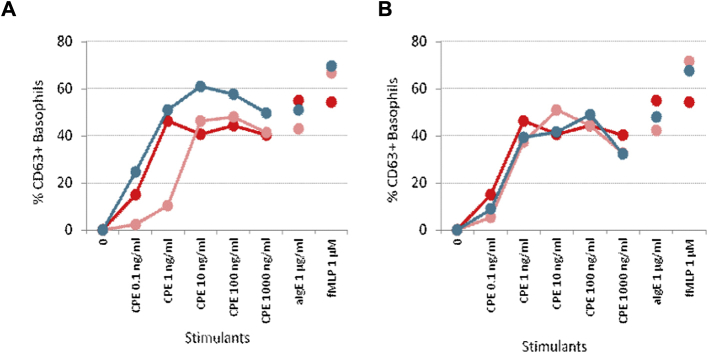


**Fig E6:**
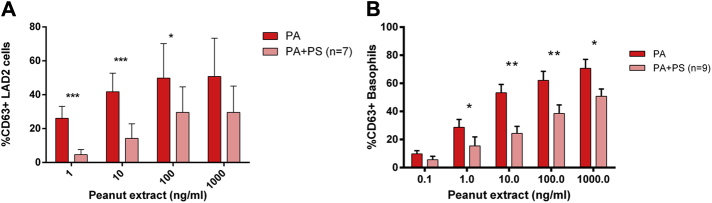


**Fig E7:**
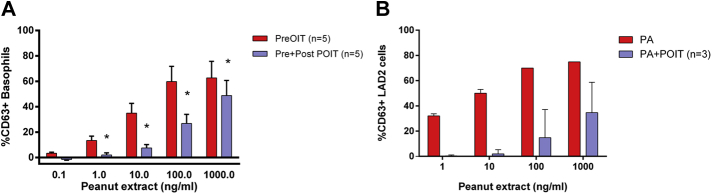


**Fig E8:**
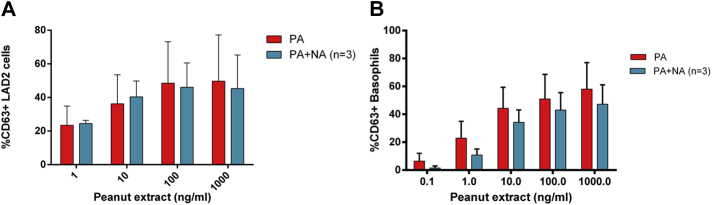


**Fig 3:**
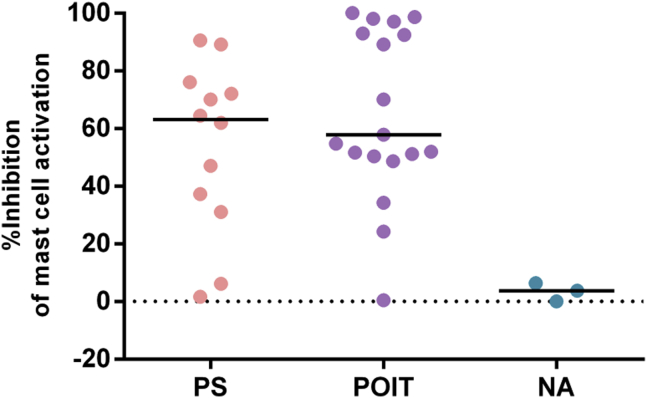
Inhibition of peanut-induced mast cell activation in the presence of plasma from PS patients with detectable P-sIgG_4_ levels (n = 12) and from patients submitted to POIT (n = 19) but not in the presence of plasma from NA subjects (n = 3). Cells were stimulated with 10 ng/mL peanut extract. Inhibition was tested against the same plasma of a patient with PA. % Inhibition = (% CD63^+^ cells sensitized with plasma from a patient with PA − % CD63^+^ cells sensitized with plasma from a patient with PA in the presence of test plasma)/% CD63^+^ cells sensitized with plasma from a patient with PA. *POIT*, Patients with PA who underwent POIT (ie, samples collected >6 months after treatment).

Taken together, the previous observations suggested that IgG_4_ was likely to have an inhibitory role over IgE. We hypothesized that this would be most relevant to PS patients with IgE directed to the major peanut allergens Ara h 1, Ara h 2, and Ara h 3. We tested the ability of plasma from PS patients with IgE directed to Ara h 1, Ara h 2, and Ara h 3 to inhibit mast cells and basophils sensitized with plasma from a reference patient with PA also sensitized to Ara h 1, Ara h 2, and Ara h 3. We used samples of patients submitted to POIT and of NA subjects as controls. Peanut-induced mast cell and basophil activation was inhibited in the presence of plasma from PS patients and in the presence of plasma from patients submitted to POIT but not in the presence of plasma from NA subjects (Fig 3 and see Figs E5-E8 in this article's Online Repository at www.jacionline.org).

### Removal of IgG_4_ antibodies partially restored peanut-induced mast cell activation

**Fig E9:**
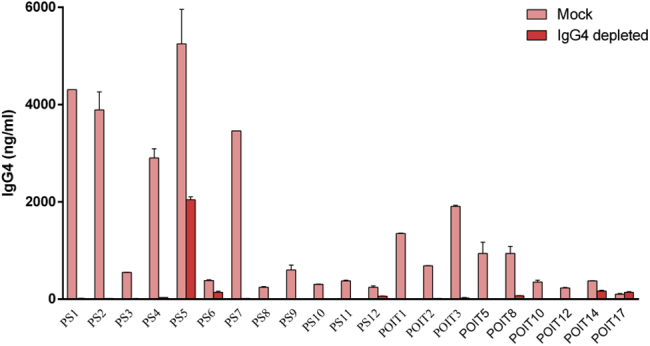


**Fig E10:**
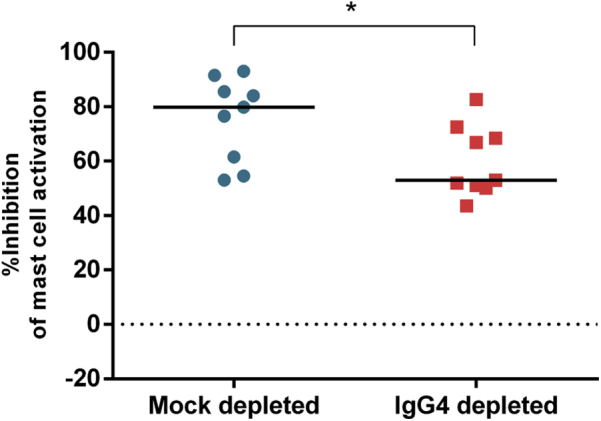


**Fig 4:**
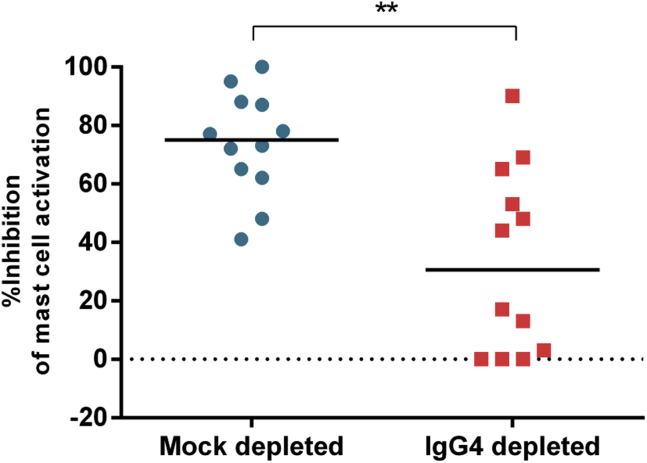
Inhibition of peanut-induced activation of mast cells sensitized with plasma from a patient with PA in the presence of mock- or IgG_4_-depleted plasma samples from peanut-sensitized tolerant patients (median inhibition, 75% vs 30%, respectively; *P* = .007, n = 12). % Inhibition = (% CD63^+^ cells sensitized with plasma from a patient with PA − % CD63^+^ cells sensitized with plasma from a patient with PA in the presence of test plasma)/% CD63^+^ cells sensitized with plasma from a patient with PA. The *P* value refers to the comparison between IgG_4_- and mock-depleted paired samples by using the Wilcoxon signed-rank test. **P < .01.

Samples from PS patients with IgE to the 3 major peanut allergens Ara h 1, Ara h 2, and Ara h 3 and with detectable P-sIgG4 levels (see Table E3 in this article's Online Repository at www.jacionline.org) showed inhibition of peanut-induced mast cell activation, as did samples from patients submitted to POIT. To confirm the role of IgG_4_ in the inhibition of peanut-induced mast cell activation, we depleted these plasma samples of IgG_4_ (see Fig E9 and Table E4 in this article's Online Repository at www.jacionline.org) and retested IgG_4_- and mock-depleted samples in the inhibition of mast cell activation assay. IgG_4_ depletion from samples from PS patients partially restored peanut-induced mast cell activation (*P* = .007, Fig 4). Reduction of mast cell inhibition was also observed when testing IgG_4_-depeleted post-POIT samples (*P* = .04, see Fig E10 in this article's Online Repository at www.jacionline.org).

## Discussion

The presence of P-sIgE does not equate to clinical peanut allergy. The majority of peanut-sensitized patients are peanut tolerant (ie, able to eat age-appropriate amounts of peanut without having an allergic reaction). It is possible that different mechanisms underpin peanut tolerance in different subgroups of PS patients. In some cases the discrepancy between IgE levels and clinical reactivity is due to differences in the specificity of IgE for peanut allergens, such as in PS patients whose IgE does not recognize any of the major peanut allergens and binds only to Ara h 8 or Ara h 9. In other cases, particularly in PS patients with IgE directed to the major peanut allergens (eg, Ara h 1, Ara h 2, and Ara h 3), which is known to be able to elicit effector cell activation and degranulation^18,19^ and would otherwise be a strong predictor of peanut allergy, inhibition of IgE by blocking antibodies might explain in part the absence of allergic symptoms after peanut ingestion. This study shows that oral tolerance to foods is associated with the presence of IgG_4_ in the context of patients who are producing IgE antibodies. Our results suggest that this might be due to a direct causal effect of IgG_4_ or, alternatively, to a related effect, such as IL-10; this might also explain why there are so many more sensitized than allergic subjects.

Using passive sensitization assays, we reproduced our previous findings in a whole-blood basophil activation assay^5^ and confirmed that the factors responsible for allergen-induced effector cell activation and unresponsiveness in patients with PA and PS patients are present in plasma. Other evidence that supports a role for plasma rather than cellular intrinsic factors in clinical reactivity to peanut is the fact that clinically determined PS patients who are able to eat peanut without any problems are often allergic to other foods and airborne allergens. This indicates that their basophils and mast cells are functional and able to respond to allergens. Experimentally, basophils from patients with PA and PS patients have similar IgE receptor expression on their surface and are able respond to other IgE-mediated stimulants apart from allergen.^20,21^ In this study basophil numbers and baseline activation were similar across groups (see Table E5 in this article's Online Repository at www.jacionline.org). Therefore intrinsic differences in cell reactivity between children with PA and PS children seem an unlikely explanation for the discrepancy between allergy and sensitization.

At the population level, median serum levels of P-sIgE were higher in patients with PA compared with those in PS patients. This has been documented in different studies and has formed the basis of the development of diagnostic cutoffs for P-sIgE levels.^22,23^ However, at the subject level, there was a great overlap between patients with PA and PS patients. In a previous study a subgroup of peanut-sensitized patients with equivocal diagnosis showed no statistically significant differences in levels of sIgE to peanut components between patients with PA and PS patients, except for Ara h 2.^5^ Patients with PA were more likely to have IgE directed to Ara h 2 alone or to Ara h 2 in combination with the other peanut major allergens compared with PS patients and had higher levels on average. However, some patients with PA did not have detectable IgE to Ara h 2, and conversely, some PS patients had Ara h 2–specific IgE levels greater than the cutoffs that have been identified as being associated with a high probability of clinical peanut allergy.^3,24,25^ Various examples of patients with PA and PS patients with the same IgE sensitization pattern to peanut allergens but with opposite clinical outcomes could be found in our cohort. Taken together, these observations confirm that the levels and specificity of P-sIgE do not account for the differences in allergic reactivity to peanut in all patients with PA and PS patients.

The hypothesis of a peanut-specific antibody counteracting the ability of P-sIgE to activate and degranulate basophils and mast cells becomes more plausible in cases in which P-sIgE levels are high and/or when directed to peanut allergens that are known to be potent elicitors of effector cell activation, such as Ara h 1, Ara h 2, and Ara h 3. In these cases IgG_4_ antibodies and possibly peanut-specific antibodies of other isotypes block IgE either by competing with IgE for binding to the peanut allergens or by inhibiting an activator response at the cellular level by co–cross-linking of IgE and IgG receptors.^26^ The 2-step experimental design used in the mast cell inhibition experiments allowed not only for a more efficient sensitization phase using plasma from patients with PA but also to reduce the effect of IgE in the blocking plasma and the effect of IgG in the sensitizing plasma. Other factors that could have influenced the results obtained with the inhibition assay are the possible effects of the generation of complement-activating (IgG_1_, IgG_2_, IgG_3_, and IgM) immune complexes and the expression of activating/inhibitory Fcγ receptors and susceptibility to complement activation products in the effector cells. The use of a whole-blood basophil activation test assay with removal of plasma before stimulation with allergen would avoid some of these issues. Ideally, we would have compared patients with PA and PS patients with the exact same levels and patterns of sensitization to different peanut allergens, namely Ara h 2 and Ara h 8, which have shown in other studies to be the main allergen in true peanut allergy and the main cause of false-positive peanut sensitization tests; however, the heterogeneity between patient groups hindered such a direct comparison.

P-sIgG_4_ levels were greater in PS patients compared with those in PA patients, but levels of IgG_4_ to peanut components were similar between the 2 groups. Also, in a previous study in children with egg allergy, specific IgG_4_ levels to ovomucoid or ovalbumin were not significantly different between children with baked egg allergy and tolerant children. Our findings regarding IgG_4_ were similar in samples from PS children and samples from patients submitted to POIT. This is consistent with various POIT studies,^8,27,28^ where IgG_4_ levels have been reported to increase substantially with treatment and are thought to have a role as a blocking antibody. In some patients with PA and NA subjects with detectable P-sIgG_4_ levels, these antibodies are likely to be directed to nonallergenic components of peanut or to different peanut epitopes of peanut allergens compared with IgE and thus have no ability to block the effect of IgE. In patients with PA, this results in IgE-mediated effector cell reactivity, whereas in NA subjects tolerance results from the absence of P-sIgE in the first instance. Furthermore, in NA subjects there is no inflammatory response against the allergens, and B cells are not affinity matured; therefore the IgG_4_ antibodies are of low affinity and not able to block IgE.

The relative values of IgG_4_ compared with IgE, rather than absolute antibody levels, are likely to be the main factor driving IgE inhibition. The IgG_4_/IgE ratio to peanut was significantly greater in PS patients compared with that seen in patients with PA, indicating that the excess in P-sIgG_4_ levels in relation to IgE could block P-sIgE and contribute to the absence of clinical reactions to peanut in PS patients. In the literature IgE/IgG_4_ ratios are usually reported and often not corrected for the fact that IgE and IgG_4_ levels are usually measured in different units (IgE in kilounits per liter and IgG_4_ in milligrams per liter).^29–31^ We have calculated IgG_4_/IgE ratios, considering the absolute amounts of these immunoglobulins in nanograms present in the serum of each patient at any given moment, which more accurately reflects the balance between the 2 immunoglobulins that potentially have opposite effects. The IgG_4_/IgE ratio to the peanut major allergens was also higher in PS patients, particularly to Ara h 2, which is believed to be the most potent elicitor of effector cell degranulation and, consequently, of allergic reactions to peanut.^19^ Our findings are consistent with previous observations in patients submitted to POIT in whom the increase in specific IgG_4_ levels was greatest for Ara h 2–specific IgG_4_ compared with IgG_4_ directed to the other peanut allergens^32^ or in whom the increase in Ara h 2–specific IgG_4_ levels matched the sensitization profile of existing IgE.^33^ These observations support the concepts that Ara h 2 is a dominant allergen in patients with PA and that IgG_4_ responses associated with clinical improvement tend to counteract existing IgE responses. They also rekindle the discussion on the usefulness of IgG_4_ assays and IgG_4_/IgE ratios for the diagnosis of food allergy; however, the design of this study does not allow a conclusion on this issue. Allergen avoidance might influence allergen-specific IgG_4_ levels and would need to be taken into consideration. Furthermore, a well-designed statistical evaluation would be required to establish the diagnostic usefulness of the differences in component-specific IgG_4_ levels between PS patients and patients with PA.

The role of IgG_4_ in IgE inhibition in samples from PS patients undergoing POIT was confirmed by depleting this antibody isotype from patients' plasma and observing an overall reduction in peanut-induced mast cell inhibition. The fact that the effect of IgG_4_ depletion was only partial in some samples suggests that other antibody isotypes could also have an IgE inhibitory effect. For example, peanut-specific IgA antibodies could contribute to the competition with IgE for binding to peanut allergens and contribute to the overall IgE inhibitory effect; however, in this population of patients, serum peanut-specific IgA levels were only detectable in a minority and did not show any differences between the PA and PS groups (data not shown). Peanut-specific IgG_1_ antibodies could also have an inhibitory role; however, we have not tested for this, which constitutes a limitation of the study.

Other factors that need to be explored to obtain a complete understanding of the mechanisms by which IgE and allergen might or might not be able to elicit effector cell activation are IgE affinity and the specific epitopes to which IgE binds.^34^ It is possible that 2 IgE molecules that are specific for the same peanut allergen recognize different epitopes in the allergen molecule or bind to the same epitope with different affinities, leading to more or less potent effector cell activation. Christensen et al,^35^ using a panel of recombinant IgE anti–Der p 2 mAbs with defined specificity, clonality, and affinity in different combinations, showed very elegantly that greater specific IgE concentrations, greater specific activity to Der p 2, and greater epitope diversity increased basophil reactivity, whereas greater affinity increased basophil sensitivity. Arguably, in individual peanut-sensitized patients the clinical phenotype is a result of the combination of different characteristics of the existing pool of P-sIgE antibodies directed to a certain allergen, as well as of the inhibitory activity of IgG_4_ and possibly other immunoglobulin isotypes. Additionally, intrinsic differences in the IgG_4_ molecule relating to affinity and epitope specificity between patients with PA and PS patients could further be responsible for inhibiting mast cell and basophil activation.^11^

Understanding why some children have low P-sIgE levels and react when exposed to peanut while others have high P-sIgE levels but are able to eat peanut without any problems might lead us to identify future therapeutic targets for children with peanut allergy. Our data also raise the question of whether the development of IgG_4_ mAbs could be used in therapeutic trials. The known anti-inflammatory properties of IgG_4_^36^ make it a particularly interesting vehicle for a biological treatment for peanut and other food allergies in the future.

**Key messages**

• At the population level, patients with PA have higher P-sIgE levels and are more likely to have IgE directed to Ara h 2 and other peanut major allergens.
• Inhibition of IgE activity by allergen-specific IgG_4_ and possibly other antibody isotypes is an alternative mechanism of peanut tolerance in patients with increased specific IgE levels to peanut and its major peanut allergens.

## Figures and Tables

**Table I:** Clinical features and serum specific IgE levels of the study population (n = 228)

	Patients with PA (n = 108)	Peanut-tolerant subjects (n = 110)		*P* value∗
		PS patients (n = 77)	NA subjects (n = 43)	
Age (y)	6.0 (5.0-10.0)	4.0 (1.5-7.5)	5 (5.0-7.0)	**<.001**
Male subjects, no. (%)	74 (68.5)	47 (61.0)	30 (69.8)	.185
Other food allergy, no. (%)	94 (87.0)	67 (87.0)	9 (20.9)	.582
Atopic eczema, no. (%)	71 (65.7)	46 (59.7)	18 (41.9)	.248
Asthma, no. (%)	43 (39.8)	14 (18.2)	8 (18.6)	**.001**
Allergic rhinitis, no. (%)	64 (59.3)	24 (31.2)	12 (27.9)	**<.001**
Pollen allergy, no. (%)	51 (47.2)	25 (32.5)	9 (20.9)	.075
Nonatopic, no. (%)	0 (0)	0 (0)	14 (32.6)	—
SPT response to peanut (mm)	9 (7-12)	4 (1-8)	0 (0-0)	**<.001**
Specific IgE (KU_A_/L)				
Peanut	13.30 (2.26-98.70)	1.83 (0.48-5.20)	0.01 (0.01-0.02)	**<.001**
Ara h 1†	0.44 (0.04-31.13)	0.11 (0.01-0.34)	0.01 (0.01-0.01)	**<.001**
Ara h 2†	6.04 (0.90-54.55)	0.08 (0.03-0.25)	0.01 (0.01-0.04)	**<.001**
Ara h 3‡	0.12 (0.02-2.41)	0.06 (0.02-0.34)	0.01 (0.01-0.01)	.075
Ara h 8§	0.08 (0.01-2.09)	0.01 (0.01-0.23)	0.01 (0.01-0.01)	**.019**
Ara h 9‖	0.01 (0.01-0.08)	0.02 (0.01-0.25)	0.01 (0.01-0.01)	.169

For continuous variables, medians and interquartile ranges are indicated.

∗ *P* values refer to the comparison between patients with PA and PS patients using the Mann-Whitney *U* test. Values in boldface indicate statistical significance.

† n = 222.

‡ n = 221.

§ n = 220.

‖ n = 219.

**Table II:** Profiles of IgE sensitization to major peanut allergens in children with PA (n = 103) and PS children (n = 76)

Profiles of sensitization	Children with PA, no. (%)	PS children, no. (%)	*P* value
Ara h 1, 2, and 3	50 (49)	18 (24)	**.001**
Ara h 1 and 2	16 (16)	0	**<.001**
Ara h 2 and 3	2 (2)	3 (4)	.355
Ara h 1 and 3	2 (2)	9 (12)	**.007**
Ara h 1 only	0	12 (16)	**<.001**
Ara h 2 only	28 (27)	16 (21)	.234
Ara h 3 only	1 (1)	3 (4)	.203
No Ara h 1, 2, or 3	4 (4)	15 (20)	**.001**

The percentage of patients per group is represented. Specific IgE levels of 0.10 KU/L or greater were considered positive. *P* values refer to the comparison between patients with PA and PS patients using the Mann-Whitney *U* test. Values in boldface indicate statistical significance.

**Table III:** Serum specific IgG_4_ levels to peanut and peanut components in patients with PA and peanut-tolerant patients (n = 101)

Specific IgG_4_ (μg/L)	Patients with PA (n = 65)	Peanut-tolerant subjects (n = 36)		*P* value∗
		PS patients (n = 27)	NA subjects (n = 9)	
Peanut	400 (160-960)	650 (430-2140)	160 (100-580)	**.023**
Ara h 1	20 (10-60)	20 (10-80)	10 (0-30)	.684
Ara h 2	30 (10-100)	10 (0-40)	10 (0-70)	**.034**
Ara h 3	40 (20-100)	80 (30-190)	30 (20-250)	.074
Ara h 8	20 (0-50)	40 (10-100)	10 (0-50)	.068
Ara h 9	10 (0-60)	30 (10-1040)	10 (0-20)	.065

Values are presented as medians and interquartile ranges.

∗ *P* values refer to the comparison between patients with PA and PS patients using the Mann-Whitney *U* test. Values in boldface indicate statistical significance.

## Supplementary data

Tables E1–E5 and Legends for Figs E1–E10
