# Supplementary material for: IgG4 inhibits peanut-induced basophil and mast cell activation in peanut-tolerant children sensitized to peanut major allergens
Source: J Allergy Clin Immunol. 2015 May;135(5):1249–56. doi: 10.1016/j.jaci.2015.01.012 (PMC4418748; doi:10.1016/j.jaci.2015.01.012)
Supplement: Tables E1–E5 and Legends for Figs E1–E10 [file mmc1.docx]

**Online Repository:**

**Table E1.** Prevalence of sensitization to the peanut components in peanut-allergic (n=103) and peanut-sensitized but tolerant (n=76) patients. Sensitization was defined as a level of specific IgE ≥0.1 KU/L. p value refers to the comparison between the two groups of patients using Fisher Exact test.

| Prevalence of sensitization | Peanut-allergic (n, %) | Peanut-sensitized tolerant (n, %) | p value |
| --- | --- | --- | --- |
| Ara h 1 | 69 (64%) | 39 (51%) | 0.096 |
| Ara h 2 | 97 (90%) | 37 (48%) | **<0.001** |
| Ara h 3 | 55 (51%) | 33 (43%) | 0.299 |
| Ara h 8 | 46 (43%) | 23 (30%) | 0.091 |
| Ara h 9 | 23 (21%) | 25 (33%) | 0.092 |
| Ara h 1, Ara h 2 and/or Ara h 3 | 100 (96%) | 61 (80%) | **0.001** |

**Table E2.** Levels of specific IgE and IgG4 to peanut in peanut-allergic patients submitted to peanut oral immunotherapy before and after treatment (n=19).

|  | Pre-POIT | Post-OIT | p value |
| --- | --- | --- | --- |
| Peanut-specific IgE (KU/L) | 27.30 (5.71; 100.0) | 10.50 (2.83; 85.90) | p=0.573 |
| Peanut-specific IgG4 (µg/L) | 580 (270; 1510) | 7200 (1210; 21200) | **p<0.001** |
| Ratio IgG4/IgE | 16.77 (4.12; 96.80) | 170.64 (30.95; 585.75) | **p=0.001** |

Median and interquartile range are represented. p values refer to the comparison between pre and post-POIT levels using the Wilcoxon Signed Ranks test.

**Table E3.** Profile of sensitization of the peanut-sensitized but tolerant patients selected for the inhibtion studies and of the peanut-allergic patients used as a reference to assess inhibition of mast cell activation. *These PS patients were also tested in the mast cell activation assay.

| Participant ID | P-sIgE (KU/L) | Arah1-sIgE (KU/L) | Arah2-sIgE (KU/L) | Arah3-sIgE (KU/L) | Arah8-sIgE (KU/L) | Arah9-sIgE (KU/L) | P-sIgG4  (µg/L) | Arah1-sIgG4 (µg/L) | Arah2-sIgG4 (µg/L) | Arah3-sIgG4 (µg/L) | Arah8-sIgG4 (µg/L) | Arah9-sIgG4 (µg/L) |
| --- | --- | --- | --- | --- | --- | --- | --- | --- | --- | --- | --- | --- |
| PS1 | 9.31 | 0.88 | 0.42 | 0.27 | 0.22 | 11.9 | 760 | 30 | 20 | 40 | 40 | 3570 |
| PS2* | 97.1 | 88.30 | 82.30 | 1.61 | 0.20 | 43.90 | 6090 | 700 | 10700 | 60 | 4 | 1040 |
| PS3* | 128 | 15.20 | 0.67 | 5.62 | 0.26 | 0.25 | 3360 | 80 | 30 | 1450 | 20 | 20 |
| PS4* | 35.7 | 0.08 | 1.84 | 0.71 | 1.01 | 8.48 | 3940 | 80 | 60 | 110 | 90 | 2770 |
| PS5 | 5.21 | 0.36 | 0.24 | 0.2 | 0.01 | 11 | 600 | 90 | 40 | 80 | 50 | 1900 |
| PS6* | 5.18 | 0.96 | 0.52 | 0.68 | 0.01 | 0.02 | 1130 | 0 | 810 | 410 | 0 | 0 |
| PS7* | 3.16 | 1.79 | 0.26 | 0.17 | 6.06 | 0.22 | 650 | 40 | 10 | 100 | 80 | 960 |
| PS8 | 0.71 | 0.13 | 0.28 | 0.12 | 0.08 | 0.1 | 70 | 0 | 0 | 0 | 0 | 0 |
| PS9 | 2.09 | 0.21 | 0.24 | 0.32 | 0.85 | 0.02 | 320 | 0 | 0 | 10 | 0 | 10 |
| PS10 | 22.2 | 11.7 | 0.59 | 1.21 | 0.01 | 0.01 | 4300 | 480 | 4720 | 620 | 0 | 0 |
| PS11 | 3.95 | 0.3 | 0.16 | 0.35 | 62.3 | 0.22 | 2440 | 130 | 30 | 1410 | 2740 | 10 |
| PS12 | 0.49 | 0.19 | 0.38 | 0.2 | 0.16 | 0.23 | 580 | 10 | 120 | 60 | 0 | 30 |
| PA | 255 | 84.4 | 125 | 1.8 | 0 | 0 | 100 | 30 | 20 | 10 | 0 | 10 |

**Table E4.** Levels of specific IgG4 to peanut in plasma samples from peanut-sensitized tolerant patients (n=12) and patients submitted to peanut oral immunotherapy (n=9) following depletion of IgG4. Peanut specific IgG4 (µg/mL) were determined by ImmunoCAP. Abbreviation: nd: not detectable

|  | Mock-depleted | IgG4-depleted |
| --- | --- | --- |
| PS1 | 0.24 | nd |
| PS2 | 0.62 | nd |
| PS3 | 0.25 | nd |
| PS4 | 0.49 | nd |
| PS5 | 0.19 | 0.03 |
| PS6 | 0.01 | nd |
| PS7 | 0.10 | nd |
| PS8 | nd | nd |
| PS9 | 0.02 | nd |
| PS10 | 0.46 | nd |
| PS11 | 0.28 | nd |
| PS12 | 0.11 | nd |
| POIT1 | 1.99 | nd |
| POIT2 | 2.29 | nd |
| POIT3 | 1.10 | nd |
| POIT5 | 0.27 | nd |
| POIT8 | 2.22 | nd |
| POIT10 | 2.31 | nd |
| POIT12 | 0.29 | nd |
| POIT14 | 1.58 | 0.05 |
| POIT17 | nd | nd |

**Table E5.** Percentage and baseline activation of circulating basophils in peanut-allergic (n=68), peanut-sensitized but tolerant (n=60) and non-sensitized non-allergic (n=41) patients.

|  | Peanut-allergic | Peanut-sensitized tolerant | Non-sensitized non-allergic | p value |
| --- | --- | --- | --- | --- |
| % of circulating basophils | 0.49  (0.36; 0.70) | 0.44  (0.34; 0.80) | 0.49  (0.38; 0.79) | 0.934 |
| %CD63+ circulating basophils | 1.35  (0.87; 1.86) | 1.02  (0.89; 1.85) | 1.1  (0.93; 1.68) | 0.637 |

**Figure legends:**

**Figure E1.** Peanut-induced activation of basophils sensitized with plasma from PA, PS and NA patients (n=15, 5 per group) after stimulation with 10ng/mL of peanut extract.

Footnote: The percentage of CD63+ basophils for each patient is expressed as the ratio between the maximal reactivity to peanut extract of the basophils sensitized with tested plasma and the maximal reactivity to peanut extract of basophils sensitized with plasma from a peanut-allergic subject used as an internal control in all experiments, to correct for the variability in cell reactivity among basophil donors. Horizontal lines represent the median for each group. p values correspond to the comparison between groups using the Mann Whitney U Test. **p<0.01.

**Figure E2.** Specific IgE to peanut and peanut components in peanut-allergic and peanut tolerant children (n=228).

Footnote: Horizontal lines represent the median for each group. p values correspond to the comparison between groups using Mann Whitney U Test.

**Figure E3.** Specific IgG4 to peanut and peanut components in peanut-allergic and tolerant children (n=101). Horizontal lines represent the median for each group. p values correspond to the comparison between patients with PA and PS patients using the Mann Whitney U Test.

**Figure E4.** Distribution of IgE and IgG4 levels in PA, PS and NA patients. PA tend to have higher levels of IgE to peanut and peanut major allergens and PS patients tend to show a predominance of IgG4 over IgE. Log base 10 of IgE and IgG4 levels are represented.

**Figure E5.** IgE was stripped of basophils from a healthy atopic donor (non peanut-allergic, no peanut-specific IgE) and the basophils were sensitized with plasma from a peanut-allergic patient (in red). Subsequently, plasma from a PS patient (in pink) or from a NA patient (in blue) were added to the basophils before stimulation with peanut extract or the positive controls (figure A). In figure B, the same steps were taken; however, basophils were washed before stimulation with peanut.

**Figure E6.** Inhibition of peanut-induced (A) mast cell and (B) basophil activation in the presence of plasma from peanut-sensitized but tolerant patients. p values correspond to the comparison between groups using the Wilcoxon Signed Ranks Test. ***p<0.001 **p<0.01 *p<0.05.

**Figure E7. A.** Peanut-induced activation of mast cells sensitized with plasma from PA patients is inhibited in the presence of post-treatment plasma from patients who underwent peanut oral immunotherapy.

**B.** Peanut-induced activation of basophils sensitized with pre-treatment plasma from patients submitted to peanut oral immunotherapy is inhibited in the presence of post-treament plasma. p values correspond to the comparison between groups using the Wilcoxon Signed Ranks Test. *p<0.05.

**Figure E8.** Peanut-induced activation of (A) mast cells and (B) basophils sensitized with plasma from peanut-allergic patients in the presence of plasma from non-sensitized non-allergic patients (n=3). Groups were compared using the Wilcoxon Signed Ranks Test but the differences were not statistically significant.

**Figure E9.** IgG4 ELISA following depletion of IgG4 from plasma samples of peanut-sensitized tolerant (PS) patients with detectable specific IgG4 to peanut and IgE to Ara h 1, Ara h 2 and Ara h 3 (n=12) and from plasma samples of patients submitted to peanut oral immunotherapy (POIT, n=9)

**Figure E10.** Inhibition of peanut-induced activation of mast cells sensitized with plasma from a PA patient in the presence of mock-depleted plasma samples or IgG4-depleted plasma samples from patients who underwent peanut oral immunotherapy (median inhibition=80% versus 52% respectively; p=0.04, n=9).

Footnote: %Inhibition = (%CD63+ of cells sensitized with PA plasma-%CD63+ of cells sensitized with PA plasma in presence of test plasma)/%CD63+ of cells sensitized with PA plasma. The p value refers to the comparison between IgG4- and mock-depleted paired samples using the Wilcoxon Signed Ranks Test *p<0.05.
